# Anti-IL5 Monoclonal Antibodies Reduce Asthma Exacerbations and Corticosteroids Dose in Three Eosinophilic Granulomatosis with Polyangiitis Case Reports

**DOI:** 10.31138/mjr.34.2.238

**Published:** 2023-06-30

**Authors:** Elpida Skouvaklidou, Maria Kipourou, Dimitrios Zisopoulos, Periklis Vounotrypidis, Konstantinos Katsoulis

**Affiliations:** 1 Respiratory Medicine Department, 424 General Military Hospital (424 GMHT), Thessaloniki, Makedonia Central, Greece,; 2 Rheumatology Department, 424 General Military Hospital (424 GMHT), Thessaloniki, Makedonia Central, Greece

**Keywords:** eosinophilic granulomatosis with polyangiitis, monoclonal antibodies, asthma, corticosteroids, case report

## Abstract

Eosinophilic Granulomatosis with Polyangiitis (EGPA) is a rare multisystem necrotizing small and medium-sized vessel vasculitis with eosinophilic adult-onset asthma as part of its spectrum. Therapeutic choices include corticosteroids, immunosuppressive agents, and novel immunomodulators. Mepolizumab and benralizumab are monoclonal antibodies targeting Interleukin-5 (IL-5), which plays a leading role in every stage of production and maturation of eosinophils and are recently undergoing evaluation and administered in steroid-dependent, relapsing and/or refractory EGPA. Herein we describe the cases of three patients with a prior EGPA diagnosis, experiencing frequent asthmatic exacerbations despite oral and inhaled corticosteroid treatment (two patients) and adverse effects of corticosteroids (one patient). Two patients are under treatment with mepolizumab and one patient with benralizumab as an add-on supplemental regimen. In our case series anti-IL5 monoclonal antibodies proved efficient asthma-controlling and corticosteroid-sparing agents.

## INTRODUCTION

Eosinophilic Granulomatosis with Polyangiitis (EGPA) is an Antineutrophil Cytoplasmic Antibody (ANCA) associated vasculitis^1^ with a prevalence of 15.27 cases per million people worldwide.^2^ The spectrum of manifestations is variable and involve multiple systems. Predominant features include adult-onset asthma, peripheral and tissue eosinophilia, and extravascular eosinophil granulomata. Extrapulmonary clinical presentation may be marked with involvement of cardiac, renal, gastrointestinal, neural system, skin and/or generalised constitutional symptoms.^1,3^ The armamentarium of EGPA treatment comprises mainly of corticosteroids and immunosuppressive agents with biological therapy being the newest option.^3^ Circulating interleukine-5 (IL-5) is increased in active EGPA and possibly interferes with production, maturation, activation, and tissue tropism of eosinophils.^4^ Anti-IL5 antibodies are under investigation for their potential therapeutic role in different disease settings. Mepolizumab, reslizumab and benralizumab target the IL-5 axis and are currently undergoing efficacy evaluation administered in steroid-dependent, relapsing and/or refractory EGPA.^3^ In accordance with the rarity of EGPA, the aim of this case series is to report three case reports diagnosed with EGPA according to the American College of Rheumatology (ACR)/European Alliance of Associations for Rheumatology (EULAR) criteria^5^ (**Table 1**) successfully treated with an anti-IL5 agent as an asthma-controlling and corticosteroid-sparing supplemental regimen.

**Table 1.** ACR/EULAR Classification criteria.

**Criteria/case**		**Case 1**	**Case 2**	**Case 3**
**Diagnosis of small or medium vessel vasculitis**		✓	✓	✓
**Alternative diagnosis**		**x**	**x**	**x**
**ACR/EULAR Classification criteria**				
**Clinical criteria**	**Score**			
Obstructive airway disease	+3	✓	✓	✓
Nasal polyps	+3	x	x	x
Mononeuritis multiplex	+1	x	x	✓
**Laboratory and biopsy criteria**	**Score**			
Blood eosinophil count ≥ 1x10^9^/lt	+5	✓	✓	✓
Extravascular eosinophilic-predominant inflammation on biopsy	+2	x	x	x
Positive test for c-ANCA or antiproteinase 3 (anti-PR3) antibodies	−3	x	x	x
Haematuria	−1	x	x	x
Total score		8	8	9

ACR: American College of Rheumatology; EULAR: European Alliance of Associations for Rheumatology; ANCA: Antineutrophil Cytoplasmic Antibody.

## CASE SERIES DESCRIPTION

### Case 1

We report the case of a 36-year-old Caucasian female with a medical history compatible with EGPA (diagnosed nine years before) and difficult to treat asthma^6^ (diagnosed two years prior to EGPA diagnosis). In October 2013 she presented in the emergency department with worsening dyspnoea and non-productive cough starting four days earlier. She had tachycardia (150beats/min), bilateral crackles on auscultation, a chest X-ray showing bilateral infiltrates and acute respiratory deficiency type I. Laboratory analysis demonstrated elevated white blood cells with neutrophilia, elevated liver enzymes, high troponin, and Pro-B-type natriuretic peptide. An emergency cardiology consultation highlighted severe left ventricle systolic dysfunction with an ejection fraction (EF) of 25% and a Pulmonary Arterial Systolic Pressure (PASP) of ≥65-70mmHg. The patient was admitted in the Intensive Care Unit (ICU). Further investigation revealed significantly elevated IgE (2800mg/dl), blood eosinophilia (6520cells/μl), bilateral ground glass opacities, pulmonary infiltrates and fibrotic bands on chest computed tomography (CT). ANCA, rheumatoid factor, antinuclear antibodies proved negative. A cardiac Magnetic Resonance Imaging (MRI) revealed diffuse subendocardial enhancement. Bronchoalveolar lavage biopsies obtained through bronchoscopy were not suggestive of eosinophilic inflammation, given that the patient was on systematic corticosteroids for several days. The patient was diagnosed with EGPA and received induction therapy as shown in **Table 2**. She gradually improved (eosinophilia resolved, EF 45-50%, PASP 25–30mmHg) and was discharged from hospital receiving the treatment shown in **Table 2**. Her stable treatment from April 2014 to September 2017 is shown in **Table 2**. In September 2017 despite receiving triple inhaled treatment and oral corticosteroids (OCS) the patient’s asthma was categorised as stage five according to Global Initiative for Asthma (GINA) with poor symptom control and frequent exacerbations (minimum four in six months) which required treatment enhancement. The patient had severe airway obstruction as shown in **Table 2** and scored seven out of 25 in Asthma Control test (ACT) questionnaire. Due to difficult-to-treat asthma,^6^ subcutaneous mepolizumab 300mg every four weeks was considered based on published data and received off label approval from the Medical Authorities for adjunctive treatment. Both physician (physical examination, spirometry) and patient-reported measures (ACT questionnaire) assessing asthma demonstrated significant improvement starting during the first months of treatment with perennially favourable outcomes throughout the five-year period of treatment. Spirometry results demonstrated an increase of Forced Expiratory Volume in One Second (FEV1) and asthma exacerbations are considerably reduced in extent and severity, with the patient experiencing a maximum of one per year. Furthermore, treatment with mepolizumab led to a gradual mycophenolate mofetil withdrawal and a step down to 2.5mg of prednisolone without EGPA re-appearance (**Table 2**). A herpes zoster incidence was reported which may be ascribed to mepolizumab and/or corticosteroid treatment but was without severe clinical impact.

**Table 2.** Induction, maintenance, and other medication at different times.

	**Case 1**			**Case 2**		**Case 3**	
**Induction medication**	**IV CYC pulse (1000mg) monthly for six months and IV MP pulses (1000 mg per day for three consecutive days)**			**x**		**IV CYC pulse (750mg) monthly for six months and IV MP pulses(1000 mg per day for three consecutive days)**	
**Maintenance medication**	**After discharge from hospital**	**Before anti-IL5 agent initiation**	**Latest follow-up**	**Before anti-IL5 agent initiation**	**Latest follow-up**	**Before anti-IL5 agent initiation**	**Latest follow-up**
OCS	PL 10mg qd	PL 10mg qd	PL 2,5mg qd	MP 16mg qd	x	MP 16mg qd	MP 4mg qd
ICS + Bronchodilators	x	BDF (640+18) mcg bid TB 2.5mcg qd	BDF (640+18) mcg bid TB 2.5mcg qd	BDF 320/9 mcg bid	BDF 320/9 mcg bid	BDP/FOR 100/6mcg bid	BDP/FOR 100/6mcg bid
MMF	x	2gr qd	x	x	x	1000mg bid	1000mg bid
AZA	x	x	x	x	x	x	X
MTX	x	x	x	x	x	x	X
RTX	x	x	x	x	x	x	X
MEP	x	x	300mg/four weeks	x	300mg/four weeks	x	X
Benralizumab	x	x	x	x	x	x	30mg/eight weeks
Other medication	*	$	$	x	x	&	&

CYC: cyclophosphamide; MP: methylprednisolone; OCS: oral corticosteroids; PL: prednisolone; ICS: inhaled corticosteroids; BDF: budesonide/formoterol; TB: tiotropium bromide; BDP/FOR: beclomethasone/formoterol; MMF: Mycophenolate mofetil; AZA: azathioprine; MTX: methotrexate; RTX: Rituximab; MEP: mepolizumab.

* ramipril 1.25mg qd, furosemide 20mg qd eplerenone 25mg qd digoxin 0,125mg qd (apart from Thursday and Sunday) carvedilol 6,25mg bid.

$ metoprolol 50mg qd, ramipril 1.25mg bid, furosemide 20mg qd, eplerenone 25mg qd.

& furosemide 20mg qd, eplerenone 25mg qd, metoprolol 25mg bid, ramipril 2,5mg qd, potassium gluconate per os 5ml bid.

### Case 2

We herein describe the case of 44-year-old Caucasian female diagnosed with EGPA one year ago. During the last year she had two emergency hospitalisations for angioedema with concomitant eosinophilia which subsided after prolonged per os corticosteroid treatment. She also developed polyarthritis and asthma. Her baseline prescribed medication (excluding acute phase treatment) to address the full spectrum of disease manifestations is shown in **Table 2**. Follow up ophthalmology examination seven months prior revealed cataract which is closely monitored. Cataract diagnosis after two long courses of systemic corticosteroids was attributed to their use and an immunomodulatory agent was considered as an add-on therapy intended to be used as a steroid sparing therapy. Mepolizumab at 300mg subcutaneous injections every four weeks was initiated. The patient had monthly re-examinations for six months in which period she achieved a complete withdrawal of corticosteroids. No exacerbations of asthma, polyarthritis or angioedema is reported, adverse effects have not occurred, and eosinophil count remained at 30 cells/mm^3^ throughout mepolizumab treatment.

### Case 3

A 30-year-old Caucasian male diagnosed with EGPA three years ago was considered for anti-IL5 treatment with benralizumab (30 mg SC every four weeks for the first eight weeks and 30mg SC every eight weeks subsequently) due to severe eosinophilic asthma with frequent exacerbations. Initial presentation of EGPA was diverse and associated with many systems. General symptoms included fatigue and weight loss and musculoskeletal symptoms consisted of thoracic and lower back pain. Eosinophilia and persistent hypokalaemia were prevalent. The spectrum of pulmonary features is characterized by lung infiltrates and asthma. Neurological involvement manifested with sensorimotor neuropathy affecting lower limbs with fluctuations in symptoms severity. Lower limbs vasculitis and purpura also occurred. Furthermore, thrombosis of left testicle and in multiple sections of renal cortex bilaterally were diagnosed. Digestive tract symptomatology, mainly abdominal pain, was frequent and led to upper and lower gastrointestinal endoscopy which revealed gastric ulcers, gastritis, and oedematous mucosa of rectosigmoid colon respectively. The patient was treated with methylprednisolone 32mg qd, esomeprazole 40mg bid, spironolactone 25mg qd azathioprine 50mg qd as indicated. In March 2020 (approximately one and half year after initial diagnosis) the patient experienced a life-threatening vasculitis flare which presented with worsening dyspnoea, orthopnoea, elevated heart rate (125beats/min) and oedematous lower limbs. Cardiology examination with heart ultrasound showed an EF of 20%. Spirometry results showed severe restriction (**Table 3**). The patient was immediately admitted to hospital and induction therapy was initiated as shown in **Table 2**. The patient showed gradual clinical improvement while cardiology follow-up examination a couple of days later revealed an EF of 48% (right ventricle) and 55% (left ventricle). Cardiac MRI revealed focal transmural enhancement of left ventricle. By the end of the sixth cycle of cyclophosphamide, the patient had an EF of 50% and his spirometry values were remarkably improved (**Table 3**) while on treatment shown in **Table 2** except for Mycophenolate mofetil (1000mg bid) which was prescribed in September 2020. The patient also experienced a gradual subjective amelioration of sensorimotor impairment until complete resolution of symptoms in September 2021, however this patient-reported finding was not ascertained by conducting an objective follow-up clinical and laboratory examination. Despite being treated with OCS the patient had recurrent asthmatic symptoms and experienced clinical and spirometric asthma exacerbations on step down of the systemic corticosteroids. In November 2021, Benralizumab was administered as supplemental therapy for asthma and proved successful in reducing asthma exacerbations and improving spirometry results. In the latest follow-up examination (November 2022) the patient had an excellent asthma control and normal spirometry values (**Table 3**) while achieving an effective tapering of methylprednisolone from 16mg to 4mg qd and zeroing this eosinophil number (**Table 2**).

**Table 3.** Spirometry tests of Cases 1 and 3.

	**Case 1**		**Case 3**		
**Spirometric values**	**Before anti-IL5 agent initiation**	**Latest follow-up**	**During severe vasculitis flare**	**Before anti-IL5 agent initiation**	**Latest follow-up**
FVC	2.42 L (74%)	3.33 L (96%)	2.35 L (42%)	4.27L (77%)	4.61 L (83%)
FEV1	1.87 L (66%)	2.30 L (80%)	2.10 L (45%)	3.87L (84%)	3.98 L (88%)
FEV1/FVC	77.3	69.1	89.4	90.6	86.3
PEF	5.47 L/s	6.04 L/s	7.6 L/s	9.92 L/s	9.9 L/s

FVC: Forced Vital Capacity; FEV1: Forced Expiratory Volume in one second; PEF: Peak Expiratory Flow.

## DISCUSSION

EGPA is a necrotising small and medium-sized vessel vasculitis with different disease phenotypes according to ANCA status.^3,7^ Symptomatology is heterogeneous with cross-sectionally considerable intra and inter patient variability. The hallmark of EGPA is prominent blood and tissue hypereosinophilia (>10% or > 1.500/mm^3^) often forming granulomata and implicated in cytotoxicity. The vast majority of patients are diagnosed with difficult-to-treat,^6^ steroid-dependent asthma with frequent exacerbations despite optimum inhaled therapy. Other respiratory signs are rhinitis, sinusitis, lung infiltrates, pleural effusion, and alveolar haemorrhage. Generalised constitutional features of weight loss, fever and myalgias are also commonly descripted. Eosinophil toxicity may affect the cardiac muscle and the digestive tract causing cardiomyopathy and/or pericarditis and mucosal inflammation respectively. Neural system involvement may take the form of sensorimotor mononeuritis multiplex or peripheral neuropathy and skin manifestations include mainly palpable purpura and less frequently ulcers, nodules or an urticaria rash. The extend of kidney impact ranges from mild urinary sediment abnormalities to renal insufficiency due to glomerulonephritis.^1,3,7^

The therapeutic array of EGPA comprises of traditional (corticosteroids and immunosuppressants), novel agents (monoclonal antibodies)^8^ and inhaled therapies for respiratory symptoms (bronchodilations and ICS).^9^ Biologic treatment is designed to target a specific pathophysiologic route implicated in disease pathogenesis. Rituximab is a monoclonal antibody that binds to the CD20 antigen uniquely present in antibody-producing B lymphocytes leading to their elimination and presumably reducing ANCA toxicity.^9^ Both mepolizumab, a humanised monoclonal antibody that targets IL-5, and benralizumab, an IL-5 receptor antagonist, neutralise IL-5 axis which participate in eosinophil production and maturation process.^9^ In the DREAM study mepolizumab was initially investigated for severe eosinophilic asthma and proved to significantly reduce asthmatic exarcebations.^10^ M.E. Wechsler et al. administered 300 mg of mepolizumab or placebo subcutaneously every 4 weeks in relapsing or refractory EGPA. The above stand-alone randomised placebo-controlled trial highlighted the increased collective remission rates and emphasized the clinician’s ability to deescalate corticosteroids dose.^11^ Mepolizumab was initially licensed to treat EGPA in USA and in September 2021 received formal administrative approval to control asthmatic symptomatology in EGPA patients in Europe. Benralizumab has been approved for eosinophilic asthma however off-label utilization in mitigating EGPA asthmatic exacerbations is lately acknowledged in a series of open label trials and case reports.^12,13^ The MANDARA trial, which is a randomised, double-blind, active-controlled 52-week study with an open-label extension to evaluate the efficacy and safety of benralizumab compared to mepolizumab and the BITE open-label study of benralizumab are two on-going studies which hope to bring new and high-quality insights into implementing benralizumab in the treatment of EGPA.^14,15^

The ACR/Vasculitis Foundation proposes a guideline for the management of ANCA-associated vasculitis; Remission treatment may be managed with corticosteroids in both life- or organ threatening and non-threatening vasculitis manifestations as high-dose monotherapy or in combination with methotrexate, azathioprine, mycophenolate mofetil, rituximab, or mepolizumab respectively. Another preferred option for induction in severe EGPA is cyclophosphamide or rituximab over mepolizumab. Tapering of corticosteroids after successful remission is individualised^16^ and should be considered due to adverse effects implicated in their usage.^8^ Remission is preferentially maintained with methotrexate, azathioprine, or mycophenolate mofetil.^16^ The above recommendations are supported by low quality of evidence and considerable variability is witnessed in real-life settings.

Our EGPA case series presents with a diversity of symptomatology with evident eosinophilic asthma in all patients. Cases 1 and 2 were treated with mepolizumab, while case 3 received benralizumab, added to their standard of care medication. Notwithstanding, their heterogeneity, all patients were naïve to any biologic therapy and responded remarkably to an anti-IL-5 agent. Anti-IL5 treatment was particularly prone to reduce asthmatic exacerbations (Cases 1,3) and had a corticosteroid-sparing ability (partial in Cases 1,3 and completely withdrawal in Case 2). Furthermore, patients were followed for a minimum of one year to a maximum of five years with continuous administration of monoclonal antibodies with no major side effects apart from one herpes zoster in Case 1. In our case series, mepolizumab was administered subcutaneously at 300mg every four weeks, however mepolizumab dose of 100mg every four weeks may also be suggested as an alternative. The above is supported by a large European multicentre observational cohort including 203 patients with EGPA divided into two subgroups receiving mepolizumab at 100 mg or 300 mg every four weeks. The two subgroups not only achieved similar response and relapse rates, but also effective control of respiratory symptoms and reduction in oral corticosteroid dose.^17^

Possible beneficial outcomes of mepolizumab treatment in EGPA’s neurologic manifestations were recently investigated with a single-arm observational study from Nakamura et al. and a case report from Kai et al. demonstrating improvement in neuropathic pain and paresthesia.^18,19^ A unique case report of EGPA with mononeuritis multiplex who did not initially respond to mepolizumab, experienced sustained resolution of mononeuritis multiplex after being treated with benralizumab.^20^ Case 3, receiving benralizumab, was the only patient in our case series with lower limbs’ sensorimotor neuropathy, however his neuropathy slowly subsided while treated with corticosteroids, cyclophosphamide, and mycophenolate mofetil before anti-IL5 agent initiation. Sustained neurologic remission was observed during benralizumab treatment.

In accordance with previous results both mepolizumab and benralizumab proved efficacious and should be considered as steroid-sparing and asthma control agents in EGPA.

The holistic pathogenesis and management of EGPA remains elusive; however, discovering specific disease-driven routes (eg, IL-5 axis) and targeting their significant components represents a major breakthrough in understanding the disease and its treatment respectively. More research is required to elucidate the indications, contraindications, precautions, dosage, and interactions of monoclonal antibodies when used in treating EGPA manifestations safely and effectively.

## CONCLUSION

In EGPA targeted blockage of IL-5 axis with mepolizumab or benralizumab poses as a viable option in mitigating asthmatic exacerbations and achieving an OCS dose reduction thought specific context of use needs to be further ascertained with high-quality randomised control trials.

## ABBREVIATIONS

ACR: American College of Rheumatology
ACT: Asthma Control Test
ANCA: Antineutrophil Cytoplasmic Antibody
Anti-PR3: Antiproteinase 3
AZA: Azathioprine
BDF: Budesonide/Formoterol
BDP/FOR: Beclomethasone/Formoterol
CT: Computed Tomography
CYC: Cyclophosphamide
EF: Ejection Fraction
EGPA: Eosinophilic Granulomatosis with Polyangiitis
EULAR: European Alliance of Associations for Rheumatology
FEV1: Forced Expiratory Volume in One Second
FVC: Forced Vital Capacity
GINA: Global Initiative for Asthma
ICS: Inhaled Corticosteroids
ICU: Intensive Care Unit
IL-5: Interleukine-5
IV: Intravenous
MEP: Mepolizumab
MMF: Mycophenolate Mofetil
MP: Methylprednisolone
MRI: Magnetic Resonance Imaging
MTX: Methotrexate
OCS: Oral corticosteroids
PASP: Pulmonary Arterial Systolic Pressure
PEF: Peak Expiratory Flow
RTX: Rituximab
PL: Prednisolone
TB: Tiotropium Bromide
